# Suppression of methylmercury-induced MIP-2 expression by *N*-acetyl-l-cysteine in murine RAW264.7 macrophage cell line

**DOI:** 10.1186/s40001-017-0287-4

**Published:** 2017-11-09

**Authors:** Juliet David, Athira Nandakumar, Muflihatul Muniroh, Suminori Akiba, Megumi Yamamoto, Chihaya Koriyama

**Affiliations:** 1 Department of Epidemiology and Preventive Medicine, Kagoshima University Graduate School of Medical and Dental Sciences, 8-35-1 Sakuragaoka, Kagoshima, 890-8544 Japan; 2 Department of Physiology, Faculty of Medicine, Diponegoro University, Tembalang Semarang, 50725 Indonesia; 3 Integrated Physiology Section, Department of Basic Medical Science, National Institute for Minamata Disease, 4058-18 Hama, Minamata, Kumamoto 867-0008 Japan

**Keywords:** Inflammation, Methylmercury, RAW264.7 cell line, *N*-acetyl-l-cysteine, Macrophage inflammatory protein-2, Keratinocyte chemoattractant, Monocyte chemoattractant protein-5

## Abstract

**Background:**

The aim of this study is to examine the inflammatory-cytokine expressions in the presence of non-cytotoxic dose of methylmercury (MeHg) in murine macrophages, which is suspected to play an important role in brain damage caused by MeHg exposure. We focused on murine macrophage inflammatory protein-2 (MIP-2), keratinocyte chemoattractant (KC), and monocyte chemoattractant protein-5 (MCP-5). MIP-2 and KC are murine functional homologues of human IL-8 and MCP-5 for human MCP-1. Furthermore, we examined the suppressive effect of *N*-acetyl-l-cysteine (NAC) on the MeHg-induced inflammatory cytokines.

**Methods:**

In a murine RAW264.7 macrophage cell line, MeHg-induced cytokine expressions were measured using real-time PCR. The suppressive effect of NAC was examined by putting it into the culture medium together with MeHg (co-treatment). In addition, pre- and post-treatment experiments were conducted, in which the cells were treated with NAC before and after MeHg exposure, respectively.

**Results:**

Exposure to a non-cytotoxic dose of MeHg up-regulated the mRNA expression of MIP-2 and MCP-5. On the other hand, KC expression was not induced in the presence of MeHg. Effect of MeHg on MIP-2 expressions was suppressed by pre-, co-, and post-treatment with NAC. However, the suppressive effect of pre-treatment was less than the post-treatment, which was as effective as co-treatment.

**Conclusion:**

In functional homologues of human IL-8, only MIP-2 expression, not KC, was activated in the presence of non-cytotoxic dose of MeHg in murine RAW264.7 macrophage cell line. The more evident inhibitory effect of NAC observed in post-treatment experiments suggests a possible involvement of intracellular activities such as antioxidant effects.

## Background

Methylmercury (MeHg) is well known to have neurotoxicity, because MeHg passes through the blood–brain barrier and accumulates in the brain [1, 2]. MeHg shows toxicological effects even at low concentrations [3]. Cellular responses to low level of MeHg exposure involve inflammatory processes. The previous studies with non-lethal doses of MeHg, concentrations as low as 1–4 μM in human astrocytoma, and 1–10 μM in macrophage cell lines suggested a possible involvement of an early inflammatory signalling [4–6]. Inflammation and subsequent degeneration of neural tissues in the central nervous system involve glial cells and peripheral macrophages, which are main sources of various pro-inflammatory cytokines and chemokines [7–9]. In brain specimens of Minamata disease patients, strong macrophage infiltration can be found in the tissue surrounding their brain lesions. In a histochemical analysis of brain tissue obtained from those patients, mercury (Hg) was detected in macrophages throughout the brain [10].

In humans, IL-8 is reported to possess a crucial role in immune responses elicited in central nervous system [11], especially in the induction of chemotaxis against its target cells (e.g., monocytes and neutrophil). U937, a human macrophage cell line, showed the up-regulation of IL-8 in response to MeHg exposure [12]. Murine macrophage inflammatory protein-2 (MIP-2) and keratinocyte chemoattractant (KC) are functional homologues of IL-8 in human [13]. MIP-2 expression was reported to be activated in the liver of MeHg-exposed mice [14]. On the other hand, there are few studies that indicate the molecular responses of MIP-2 and KC against the direct exposure to MeHg in vitro.

Monocyte chemoattractant protein-1 (MCP-1) is also suspected to work as an alert system in response to MeHg exposure in the brain on the basis of experimental findings using mice whose murine MCP-1 homologue was knocked out [15]. In addition, at a non-cytotoxic concentration of 4 µM MeHg, Muniroh et al. [16] observed a significant induction of MCP-1 expression in U-87 MG cells, a model system cell line of human astrocytes. Murine MCP-5 was reported to be the most homologous chemokine to human MCP-1 [17, 18]. In fact, MCP-5 was structurally more similar to MCP-1 than JE, the putative murine homologue of MCP-1 [18]. However, there are no reports concerning the effect of MeHg on MCP-5 expression.

*N*-acetyl-l-cysteine (NAC), the acetylated precursor of l-cysteine, is a sulfhydryl-containing antioxidant, which has been used for the treatment of heavy metal toxicity and can act as an antiinflammatory agent [19]. It reduces reactive oxygen species levels by raising intracellular glutathione concentrations and/or playing directly as a free radical scavenger. NAC was used to modulate inflammatory pathways in peripheral and central nervous system and cytokine levels in neuropsychiatric disorders [19]. Furthermore, NAC was reported to act as a chelating agent for mercury and accelerates urinary excretion of MeHg in mice [20]. It should also be noted that a study has shown that NAC suppressed the MeHg-activated MCP-1 and IL-6 expressions in U-87 MG cell line [18], and IL-6 and IL-8 expressions in U937 macrophages [12].

Based on above background, we investigated the inflammatory responses to the non-cytotoxic concentration of MeHg, focusing on MIP-2, KC, and MCP-5 expressions as functional homologues of IL-8 and MCP-1 in mice. To expand the role of these genes for understanding the toxicity of MeHg in human, further in vivo mechanistic analysis using rodents will be necessary. In addition, we examined the suppressive effect of NAC on the MeHg-induced inflammatory cytokines.

## Methods

### Cell culture

A murine macrophage cell line, RAW264.7, was used in this study (Sumitomo Dainippon Pharma, Osaka, Japan). The cells were cultured in Dulbecco’s modified Eagle’s medium (DMEM) (Sigma-Aldrich, MO, USA) with 1% l-glutamine (Sigma-Aldrich, MO, USA), penicillin (100 U/mL), streptomycin (100 µg/mL) (Sigma-Aldrich, MO, USA), and 10% heat-inactivated fetal bovine serum (FBS) (Nichirei Biosciences, Tokyo, Japan) at 37 °C in a humidified incubator (5% CO_2_). An initial concentration of 2 × 10^4^ cells/mL was used for each experiment.

### Cytotoxicity test

Stock solution of MeHgCl (10 mM) (Tokyo Chemical Industry, Tokyo, Japan) was dissolved in Dulbecco’s PBS (Sigma-Aldrich, MO, USA) with l-cysteine (Hg:Cys = 1:1) and kept at − 80 °C until use. The stock was diluted with culture medium just before being added to the cells. NAC (Wako Pure Chemical Industries, Osaka, Japan) was dissolved in FBS-free DMEM, and the pH was adjusted to 7.4 with sodium hydroxide (NaOH).

For cytotoxicity assays, the cells were cultured for 24 h in 96-well plates and incubated with MeHg (0.1–100 µM) for 24 h. Cell toxicity was assayed using the WST-8 Cell Counting kit, according to the manufacturer’s protocol (Wako Pure Chemical Industries, Osaka, Japan). In brief, the cells were washed, and Hank’s balanced salt solution (100 µL) and WST-8 solution (10 µL) were added to each well and kept for incubation at 37 °C in 5% CO_2_. The colour was quantified within 1–2 h by a micro-plate spectrophotometer (TriStar LB941, Berthold Technologies, Germany) at 450 nm absorbance. Culture medium was used to adjust the absorbance values of the sample. Mean values and standard errors (SEs) were obtained from the results of four experiments.

### Treatments of MeHg and NAC

Non-cytotoxic concentrations of MeHg (0.5 or 2 µM) and NAC (1 or 20 mM) were used for further experiments. To examine the suppressive effects of NAC on MeHg-induced MIP-2, KC, and MCP-5 up-regulations, the following protocols were used:

#### Pre-treatment experiment

After 23 h of cell culture, cells were pre-incubated with NAC for 1 h, and the remaining NAC, if any, was washed out with a double volume of culture medium. Then, cells were incubated with the medium containing MeHg for 3 h. The incubation time of NAC was determined based on prior experiments.

#### Co-treatment experiment

After 24 h of cell culture, cells were treated with the medium containing both NAC and MeHg for 3 h, which was prepared and kept at the room temperature for 30 min in advance.

#### Post-treatment experiment

After 24 h of cell culture, cells were treated with the medium containing MeHg. Medium was washed out after 3 h of MeHg exposure and then NAC was added. Cells were harvested 30 h after the starting of cell culture.

### Analysis of mRNA expression

Total RNA from the cells was extracted using an RNeasy Plus Mini kit (Qiagen), which eliminates genomic DNA contamination. Then, cDNA was synthesized from total RNA (600 ng), using QuantiTect Reverse Transcription (Qiagen) and stored at − 80 °C until use. The samples were prepared in three independent experiments. The mRNA expressions of cytokine genes were quantified with LightCycler instrument (Roche Diagnostics Japan, Tokyo, Japan), using the LightCycler FastStart DNA Master^PLUS^ SYBR Green I (Roche Diagnostics) following the manufacturer’s instructions. The primers for each gene were designed and synthesized on the basis of respective information in NCBI, using the software of Primer3 (http://frodo.wi.mit.edu/primer3/), and the target regions were 80–300 bp in length (Sigma-Aldrich Japan, Hokkaido).

PCR amplification was conducted as reported in the previous studies [21, 22] in a total volume of 20 µL containing cDNA and each primer (0.5 µM). Forward (F) and reverse (R) primers were:

β-actin, 5′-*CGTGCGTGACATCAAAGAGAAG*-3′ (F), 5′-*ATGCCACAGGATTCCATACCC*-3′(R);

KC, 5′-*AGAACATCCAGAGCTTGAAGGTGTT*-3′(F), 5′-*GGACACCTTTTAGCATCTTTTGGACA*-3′(R); MIP-2, 5′-*AAGTTTGCCTTGACCCTGAA*-3′(F), 5′-*AGGCACATCAGGTACGATCC*-*3′*(R) and MCP-5, 5′-*TGGACCAGATGCGGTGAGC*-3′ (F), 5′-*GGCTGCTTGTGATTCTCCTGTAG*-3′(R), respectively. The DNA polymerase was activated by initial denaturation at 95 °C for 10 min, and 45 amplification cycles were carried out. The cycle consists of denaturation at 95 °C for 10 s, annealing at 60 °C for 10 s, and extension 72 °C for 15 s. The fluorescent product was detected at the end of the extension period. All PCR assays were performed three times independently.

The data were analyzed using the Light Cycler analysis software version 4.1 (Roche Diagnostics Japan, Tokyo, Japan). Amplification specificity was confirmed by melting curve analysis of the PCR products. Threshold cycle (*C*t) values of the target genes were normalized to the expression of the β-actin gene as an internal control gene. Unstimulated RAW264.7 cells harvested in 24 h were used as a standard, and unstimulated cells harvested after 30 h as a negative control. The relative expression in each sample to that of the control sample was calculated according to the 2^−ΔΔCT^ method [23].

### Statistical analysis

Mean values and corresponding SEs were calculated using the Stata 14.0 Software (StataCorp LLC, Texas, USA). The *P* value for the difference between two groups was calculated by the Mann–Whitney U test, and a non-parametric test for trend across ordered groups was performed. The level of significance was indicated by *P* < 0.05.

## Results

### Cytotoxicity of MeHg and NAC in RAW264.7 macrophage cell line

**Fig. 1:**
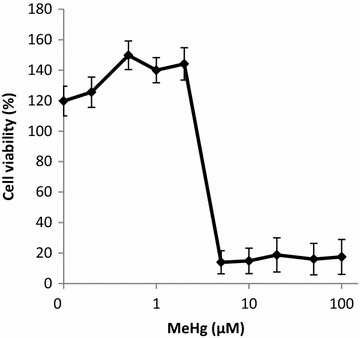
Cell viability after exposure to different doses of MeHg. MeHg (0.1–100 µM) exposure on RAW264.7 cell line and the cell viability by WST-8 cell counting Assay. Values represent the mean ± standard error of four experiments

Cytotoxicity of MeHg on RAW264.7 cells was assayed after incubating cells for 24 h in the presence or absence of MeHg. As shown in Fig. 1, the maximum non-cytotoxic dose of MeHg was 2 µM. Therefore, for cytokine-induction experiments, the doses of 0.5 and 2 µM were used. NAC was not cytotoxic up to 50 mM (data not shown). On the basis of these results, 1 mM and 20 mM of NAC were selected for further experiments.

### Effect of MeHg on mRNA expressions of MIP-2, KC, and MCP-5

**Fig. 2:**
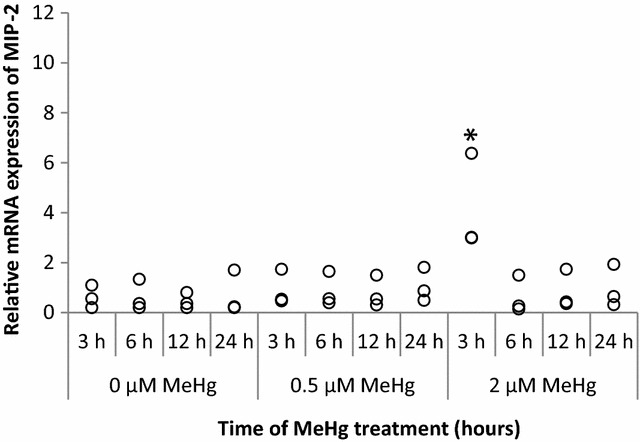
MIP-2 mRNA expression at 3, 6, 12, and 24 h after MeHg exposure. RAW264.7 cells were treated with 0 µM (control), 0.5, 2 µM of MeHg, and mRNA expression levels were analyzed by real-time PCR. Values were normalized to the expression of β-actin and it represents three independent experiments. MIP-2 mRNA expression induced by MeHg treatment alone is compared with control and the significant value is labelled as "*"

As shown in Fig. 2, MIP-2 mRNA was detected initially (in the absence of MeHg in the medium) in RAW264.7 cells. When calculating relative gene expressions, we used this initial value at time 0 as the reference. In response to 2 µM of MeHg, MIP-2 mRNA expression was significantly induced within 3 h (*P* = 0.0495). Activation of MIP-2 mRNA expression declined after 6 h of MeHg exposure. No significant expression was observed at 6 h or after. In the lower dose, no evident up-regulation was found.

**Fig. 3:**
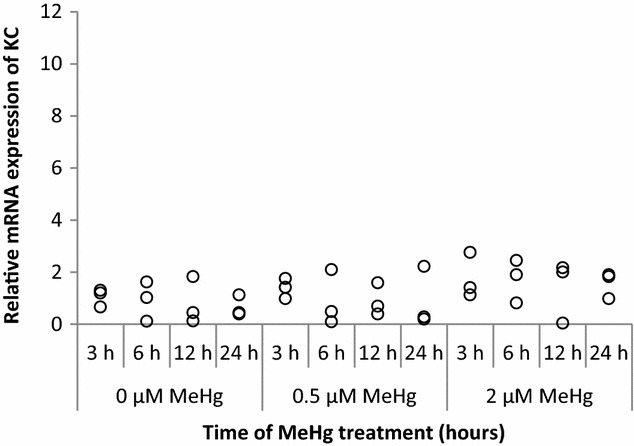
KC mRNA expression at 3, 6, 12, and 24 h after MeHg exposure. RAW264.7 cells were treated with 0 µM (control), 0.5, and 2 µM of MeHg, and mRNA expression levels were analyzed by real-time PCR. Values were normalized to the expression of β-actin and it represents three independent experiments

KC mRNA expression was not significantly up-regulated by 2 µM of MeHg (Fig. 3). When we treated the cells with lipopolysaccharide (LPS) to confirm the KC expressions in RAW264.7 cells, we could detect the KC up-regulation at 3 h (data not shown).

**Fig. 4:**
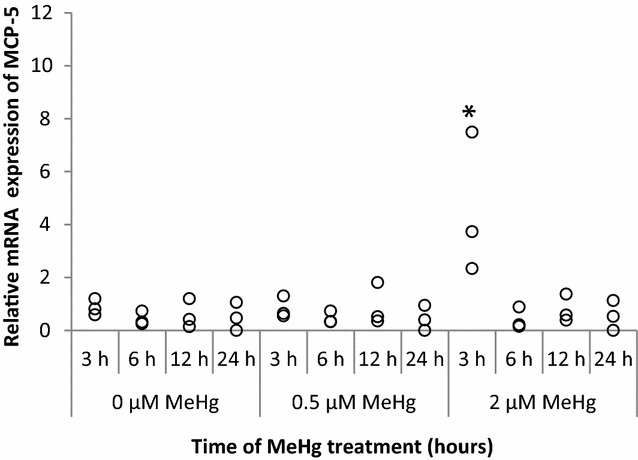
MCP-5 mRNA expression at 3, 6, 12, and 24 h after MeHg exposure. RAW264.7 cells were treated for 3, 6, 12, and 24 h with 0 µM (control), 0.5, and 2 µM of MeHg and mRNA expression levels were analyzed by real-time PCR. Values were normalized to the expression of β-actin and it represents three independent experiments. MCP-5 mRNA induced by MeHg treatment alone is compared with control and the significant value is labelled as "*"

MCP-5 expression was significantly induced within 3 h in response to 2 µM of MeHg (*P* = 0.0495). Activation of MCP-5 mRNA expression decreased after 6 h of MeHg treatment. In the lower dose, no obvious up-regulation was observed (Fig. 4).

### Effect of different timing of NAC treatment on mRNA expression of MIP-2

**Fig. 5:**
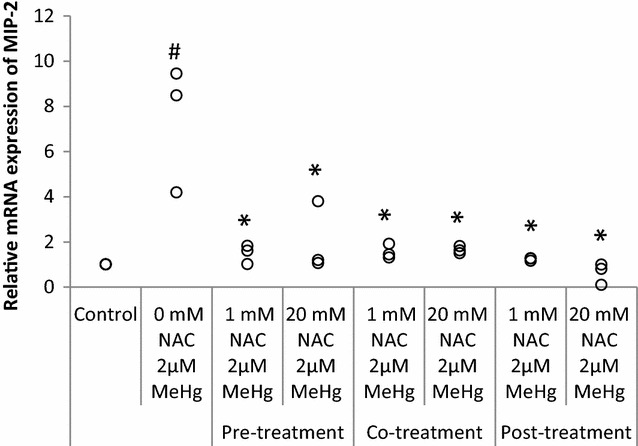
Effect of NAC to suppress MeHg-induced MIP-2 mRNA expression. RAW264.7 cells were treated with 1 or 20 mM NAC at 1 h before (pre-treatment), with (co-treatment), or 1 h/3 h after 2 µM MeHg (post-treatment) for 3 h of incubation. mRNA expression levels of MIP-2 were analyzed by real-time PCR values which were normalized to the expression of β-actin and it represents three independent experiments. MIP-2 mRNA expression induced by MeHg treatment alone is compared with control, *P* = 0.0369 (labelled as ^#^), MeHg + NAC treatment (1, 20 mM) effects are compared with the MeHg treatment alone and the significant values (*P* = 0.0495) were shown in the figure (labelled as *)

Figure 5 shows the effect of NAC pre-treatment, co-treatment, and post-treatment on MIP-2 mRNA expression induced by MeHg exposure. For this experiment, MIP-2 was selected, because its response to MeHg was remarkable. The NAC treatment decreased MeHg-induced MIP-2 expression in pre-treatment (*P* = 0.020), co-treatment (*P* = 0.020), and post-treatment (*P* = 0.020) when the NAC treatment groups and non-treatment groups were compared. In pre-treatment, MeHg-induced MIP-2 expressions were significantly inhibited by NAC regardless of dose (*P* = 0.0495 for both doses). In co-treatment, regardless of NAC dose, MIP-2 gene expressions were significantly suppressed (*P* = 0.0495 for both doses). In post-treatment, NAC also inhibited MIP-2 expressions significantly (*P* = 0.0495 for both doses). Significant dose-dependent reduction of MIP-2 was observed only in the post-treatment (*P* for trend 0.017).

The post-treatment of NAC was more effective than co-treatment regardless of dose (*P* = 0.0495 for both doses) and post-treatment of NAC was more effective than pre-treatment only when NAC dose was 20 mM (*P* = 0.0495).

## Discussion

In this study, exposure to non-cytotoxic doses of MeHg (2 µM) up-regulated MIP-2 and MCP-5 mRNA expressions in RAW264.7 macrophage cell line. To our knowledge, the present study is the first report regarding the activation of MIP-2 and MCP-5 expression by MeHg in vitro. Note that murine MIP-2 and KC are functional homologues of human IL-8 [15, 16]. The MIP-2 but not KC expression was significantly activated by MeHg, indicating that only the MIP-2, as a part of human IL-8 homologue, mainly plays a role in inflammatory response against MeHg exposure in murine macrophages. MIP-2 was reported to be more potent than KC in leukocyte recruitment, endothelial cell chemotaxis [24, 25], and cyclophilin-A-induced neutrophil migration [26]. In neutrophil emigration in cremaster muscle, MIP-2 and KC did not differ significantly [27, 28]. Some studies have proved that MIP-2 alone can recruit chemotactic cells, but when both MIP-2 and KC are present, the recruitment is maximal. On the other hand, Tanimoto et al. [26] reported that, on the basis of different studies, MIP-2 and KC also complemented each other in their roles and functions. The expression patterns of KC and MIP-2 are tissue-specific, and therefore, their roles in chemotactic cells recruitment are different from tissue to tissue [29, 30]. The functional regulation of IL-8 in the presence of MeHg was different between human and mice. These results will be useful for further in vivo functional analysis of these gene expressions using mice.

Muniroh et al. [4] and Yamamoto et al. [5] also showed suppressive effects of NAC on MeHg-induced cytokine expressions. The importance of the antioxidant mechanism of NAC and its antiinflammatory effect is emphasized in reference to a report in which NAC inhibits LPS-induced cytokine activation [30]. In the present study, the up-regulation of MIP-2 in response to exposure to MeHg was suppressed by NAC pre-, co-, and post-treatments. In the pre-treatment experiment, the medium containing NAC was washed out before the MeHg exposure, and hence, the NAC could only act intracellularly. In the co-treatment experiment, the medium was mixed previously with NAC and MeHg. If the co-treatment was most effective, it suggests a strong involvement of chelating effect of NAC in the medium. In the post-treatment, the medium containing MeHg was washed out before addition of NAC into the medium. Since the post-treatment was as effective as co-treatment, there is no evidence to indicate an extracellular effect such as a chelating effect. The inhibitory effects of NAC shown in pre- and post-treatments indicate the involvement of intracellular activities, including antioxidant effects.

## Conclusion

In murine RAW264.7 macrophage cell line, the MIP-2, functional homologue of IL-8, was mainly involved in the inflammatory response induced by MeHg exposure. The MCP-5, a murine homologue of human MCP-1, was also involved in such an inflammatory response. The remarkable suppressive effect of NAC on MeHg-induced inflammatory-cytokine expressions observed in post-treatment experiments would suggest a possible involvement of intracellular activities such as the antioxidant effects.

## Abbreviations

MeHg: methylmercury
MIP-2: macrophage inflammatory protein-2
KC: keratinocyte chemoattractant
MCP-5: monocyte chemoattractant protein-5
NAC: *N*-acetyl-l-cysteine
MCP-1: monocyte chemoattractant protein-1
DMEM: Dulbecco’s modified Eagle’s medium
LPS: lipopolysaccharide
